# Invasive breast cancer following bilateral subcutaneous mastectomy in a *BRCA2 *mutation carrier: a case report and review of the literature

**DOI:** 10.1186/1477-7819-3-52

**Published:** 2005-08-04

**Authors:** Lidia Kasprzak, Benoit Mesurolle, Francine Tremblay, Maria Galvez, Fawaz Halwani, William D Foulkes

**Affiliations:** 1 Department of Medicine, McGill University Health Centre, Montreal, Canada; 2 Department of Radiology, McGill University Health Centre, Montreal, Canada; 3 Department of Surgery, McGill University, Montreal, Canada; 4 Department of Pathology, McGill University, Montreal, Canada; 5 Program in Cancer Genetics, Departments of Oncology and Human Genetics, McGill University, Montreal, Canada

## Abstract

**Background:**

Primary prevention of breast cancer through prophylactic mastectomy can reduce the risk of malignancy in high-risk individuals. No type of mastectomy completely removes all breast tissue, but a subcutaneous mastectomy leaves more tissue *in situ *than does a simple mastectomy.

**Case presentation:**

We report a case of invasive breast cancer in a *BRCA2*-positive woman 33 years after bilateral subcutaneous mastectomy. To our knowledge, only one case of primary breast cancer after prophylactic mastectomy in a *BRCA1*-positive patient has been reported in the literature and none in *BRCA2*-positive individuals.

**Conclusion:**

Careful documentation and long follow-up is essential to fully assess the benefits and risks of preventive surgical procedures in *BRCA1 *and *BRCA2 *mutation carriers.

## Background

The risk of breast cancer in women who inherit a germline mutation in the *BRCA1 ge*ne can be as high as 20% by the age of 40 and 50% by the age of 50 [1] and as high as 13% by the age of 40 and 60% by the age of 50 in *BRCA2 *mutation carriers [2]. These estimates apply to individuals who belong to very high risk, multiple-case breast cancer families. Prophylactic surgery to reduce cancer risk remains an option for carriers of *BRCA *gene mutations; however, its efficacy is likely to depend on the ability to remove nearly all breast tissue. Different surgical procedures, (subcutaneous or a simple mastectomy), limited patient follow-up and lack of adequate control population confound the numerous studies on this subject. Only three reports have specifically addressed the extent of risk reduction by prophylactic breast removal in *BRCA1 *and *BRCA2*-positive individuals [3-5]. Most surgeons believe that subcutaneous mastectomy (SCM) is not optimal for prophylaxis because a substantial amount of breast tissue remains in the nipple-areola complex and on the skin flaps, and therefore it has fallen into disuse. Here, we report a case of breast cancer occurring following a SCM. A literature review using PubMed, searching from 1994 to the present, revealed this to be the first reported case of breast cancer occurring post-SCM in a carrier of a *BRCA2 *gene mutation. We discuss the possible implications of this seemingly uncommon finding.

## Case Presentation

**Figure 1:**
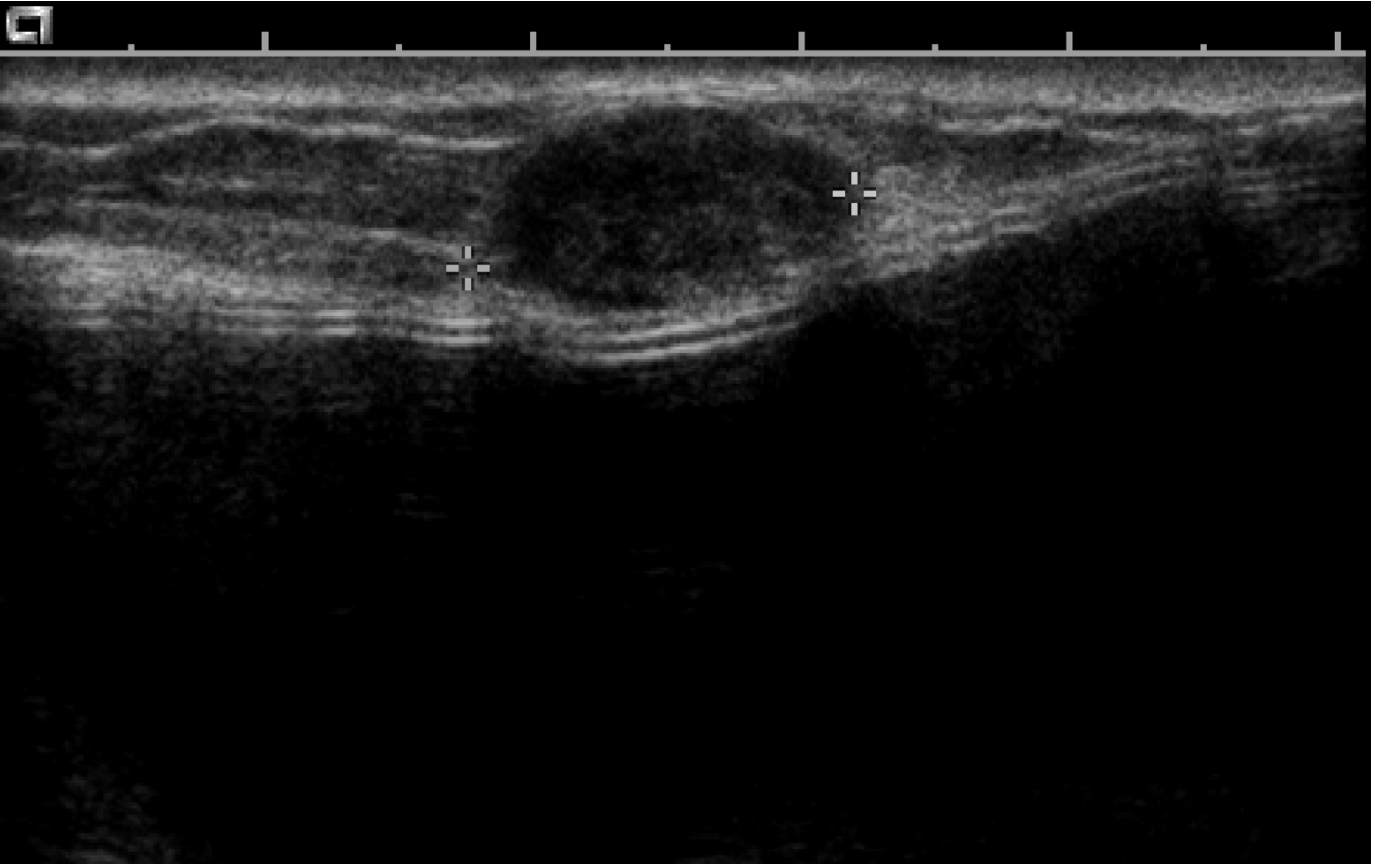
Sonographic examination: 15 mm hypoechoic solid, non-calcified circumscribed mass, with a thin echoic rim, benign in appearance, located in subcutaneous fat at 9 o'clock in the left breast.

**Figure 2:**
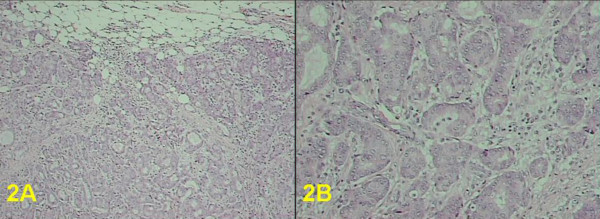
photomicrograph 2A) Invasive ductal carcinoma, apocrine type: tumor exhibits an irregular invasive border and forms glandular structures. Hematoxylin and eosin stain, original magnification × 40. 2B) Invasive ductal carcinoma, apocrine type: cytological characteristics of intermediate nuclear grade, prominent nucleoli, and eosinophilic granular cytoplasm. Hematoxylin and eosin stain, original magnification × 100.

A 49 year old G3P2A1 presented in 2002 with a six month history of a painless lump in her inner left breast. She had undergone bilateral SCM with immediate implantation of silicone prostheses at the age of 16 due to extensive fibrocystic breast disease with adenosis. On physical examination a firm, mobile, 1.5 cm nodule was palpated superficially. There was no associated skin retraction or thickening. No enlarged abnormal nodes were palpable. We performed mammographic and sonographic examinations. Mammography, of limited value because of the previous SCM, did not show any obvious abnormality. Sonographic examination revealed a 1.5 cm in size, hypoechoic, solid, non-calcified, circumscribed mass, grossly ovoid with a thin echoic rim, located in the subcutaneous fat at 9 o'clock in the left breast (Figure 1). Minimal vascular pole was identified on conventional color sonography. The imaging findings appeared to indicate a benign nature of the lesion; however, based on the family history, patient age, and the recent occurrence of the nodule, excisional biopsy was performed. An infiltrating ductal carcinoma, apocrine type, grade 2 of 3 of modified Bloom and Richardson, with associated ductal carcinoma *in situ*, cribriform type, nuclear grade 2 of 3, occupying 10% of tumor mass (Figures 2A and 2B) was identified. Estrogen receptor status was strongly positive on most malignant cells, progesterone receptor moderately positive on 50% of malignant cells and HER-2/neu was negative (score 0) with no membrane staining of malignant cells. There was no evidence of malignancy in 23 lymph nodes examined following the left axillary contents dissection.

**Figure 3:**
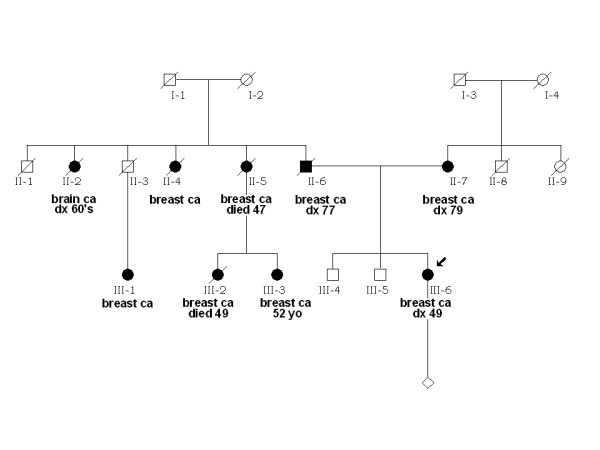
Pedigree of the family with germline *BRCA2*: 6503delTT mutation. All individuals affected with cancer (ca) are depicted by filled-in symbols. Individual ID numbers, age at the time of diagnosis (dx) and/or death are below each symbol.

Subsequently, the proband was referred for genetic counseling and found to carry *BRCA2*: 6503delTT, a mutation previously described in the French Canadian population [6]. Family history (Figure 3) was significant for breast cancer in proband's father (II-6) who was diagnosed at the age of 77 and mother (II-7) diagnosed at 79. A 52 years old paternal cousin (III-3), also affected with breast cancer, was previously identified as a *BRCA2 *mutation carrier at another institution. Of note, individual III-3's mother (II-5) and sister (III-2) both had breast cancer and died at 47 and 49 years of age, respectively. There were only two paternal aunts known to have been affected with breast cancer at the time of patient's bilateral SCM.

Given the diagnosis of invasive breast cancer and *BRCA2 *mutation carrier status, our proband opted for prophylactic bilateral salpingo-oophorectomy with hysterectomy, as well as removal of the nipple-areola complex along with remaining breast tissue. The pathology examination identified the presence of bilateral adnexal stromal hyperplasia, several leiomyomata in the myometrium as well as simple ductal epithelial hyperplasia without atypia in the mastectomy specimen. There was no evidence of malignancy.

## Discussion

Currently available management strategies for women who carry inherited predisposition to develop breast cancer are limited given the lack of prevention methods with proven efficacy. Furthermore, there have been no prospective, controlled trials of the breast cancer risk reduction associated with bilateral prophylactic mastectomy. Such studies are unlikely to take place due to ethical and practical considerations. Prophylactic mastectomy, whether subcutaneous or total, significantly reduces, but does not eliminate, the risk of breast cancer in high-risk individuals.

Several studies showed that breast reduction procedures substantially lower the risk of breast cancer. Recently, Brinton *et al *[7] confirmed that the magnitude of cancer risk reduction is directly related to the amount of tissue removed during the operation. With a SCM, the nipple-areola complex is preserved and some of the underlying breast tissue remains on the skin flaps. When SCM is intended as prophylaxis against breast cancer, the surgeon's aim is to remove as much tissue as possible. It is plausible that a less thorough removal of glandular tissue may have taken place given the indication for surgery in our patient's case. It is generally agreed that the prophylactic nature of bilateral mastectomy in an unaffected *BRCA1 *or *BRCA2 *mutation carrier calls for the most complete breast tissue removal. This viewpoint makes SCM a less desirable choice. Skin-sparing mastectomy [8] could be seen as a partial compromise and appears to be an increasingly popular option for women at high risk. More recently, geneticists have questioned the rejection of simple SCM as a viable procedure in such women. It is argued that the magnitude of the risk reduction offered by SCM, when combined with its greater cosmetic acceptability, is sufficient to keep this option available to women [9].

There are numerous reports in the literature describing the occurrence of breast cancer after SCM [10,11]. Subsequently, the perception exists that SCM fails to eliminate the risk of breast cancer. Although the extent of risk reduction achieved by SCM is limited given that about 5–10% of the mammary tissue remains *in situ*, it is thought to be of the order of >85% [4,12]. As stated above [9], at this level of risk reduction, SCM would have a greater effect on breast cancer rates in *BRCA1/2 *carriers than would total mastectomy if at least 50% of *BRCA1/2 *carriers chose preventive SCM. Currently, preventive bilateral total mastectomy rates are about 20% in most populations.

The first retrospective study of efficacy of prophylactic mastectomy carried out by Hartmann *et al *[12] included 18 subjects later confirmed to be carriers of deleterious mutations in the *BRCA *genes but, unfortunately, it had insufficient statistical power to detect a difference in the risk reduction between total and SCM. In this cohort, all breast cancers (n = 7) were diagnosed in women who had undergone SCM (total of 950). None were known to be *BRCA1/2 *mutation carriers. Of the seven cases, only one occurred in the nipple-areolar area. Not surprisingly, the majority of the high-risk women (n = 17) described in the subsequent report [3] underwent SCM. After a median of 13.4 years of follow-up, none of the *BRCA1/2 *germ-line mutation carriers has developed breast cancer. The authors concluded that at least 90% risk reduction could be expected among women with confirmed *BRCA *mutation status following prophylactic bilateral SCM. Meijers-Heijboer *et al *[4] report the initial results of a prospective study of 76 women with deleterious *BRCA1 *or *BRCA2 *mutations who chose to undergo bilateral simple mastectomy and no breast cancers were observed after a mean follow-up of 2.9 ± 1.4 years. The Prevention and Observation of Surgical End Points (*PROSE*) Study Group findings [5] support the notion that bilateral mastectomy results in approximately 90% breast cancer risk reduction. Of 105 *BRCA *mutation carriers, only two women (1.9%) developed breast cancer 2.3 and 9.2 years after SCM. The first breast cancer case was diagnosed at the age of 28 years in the *BRCA2 *mutation carrier who presented with a palpable axillary mass at 27 months post-SCM. Subsequently, metastatic adenocarcinoma in an axillary lymph node was identified and it was most likely consistent with a primary breast cancer already present at the time of SCM. It is important that this case is not considered as a failure of SCM, and it should be therefore classified as a recurrence which would have likely taken place despite the surgery. The second breast cancer case occurred in a *BRCA1 *carrier at the age of 41 years.

When hereditary predisposition to breast cancer is being assessed, it is important to consider the impact of the age-related penetrance of the *BRCA1 *and *BRCA2 *genes. *BRCA1 *has a higher penetrance than *BRCA2 *in the pre-menopausal years [1]. The benefits of preventive surgery will be proportionally greater for an older *BRCA2 *carrier than an older *BRCA1 *carrier, and therefore if a *BRCA2 *carrier discovers her mutation status when she is peri- or post-menopausal, the potential benefits of preventive mastectomy should not be understated, as the breast cancer risks do not significantly diminish following menopause.

In our review of the literature (see Table 1), only one primary breast cancer has been reported to occur in a cohort of 207 *BRCA1/2 *mutation carriers who opted for preventive surgery. It could be argued that the efficacy of the bilateral total mastectomy has not been studied adequately in the high-risk individuals to prove its absolute superiority over SCM. The total number of the *BRCA1 *and *BRCA2 *carriers who have undergone this type of mastectomy is relatively small and the mean follow-up is rather short. Furthermore, the presence of a microscopic primary lesion at the time of surgery may result in subsequent recurrence that would be impossible to differentiate from a new primary breast cancer. Based on the above data, the risk-reducing effect of SCM should not be ignored when presenting prophylactic mastectomy options to women at high risk who find total mastectomy unacceptable and would not otherwise have considered surgical prevention. Nevertheless, the lack of popularity of this procedure among surgeons will likely limit its use.

**Table 1:** Studies assessing efficacy of bilateral prophylactic mastectomy (PM) in *BRCA1 *and *BRCA2 *carriers

	Hartmann *et al *[3]	Meijers-Heijboer *et al *[4]	Rebbeck *et al *[5]
Study recruitment centers	USA	Netherlands	USA, Canada, UK, Netherlands
Median follow-up (yrs)	13.4	2.8	5.5
Mean age at surgery (yrs)	39	36	38
Number of PM patients	18	76	105
Number of controls	-	63	378
Type of study	Retrospective cohort	Prospective cohort	Case-control
Primary invasive breast cancer			
- in cases (after PM)	0	0	1 (***BRCA1***carrier)
- in controls	-	8 (13%)	184 (49%)

The existing literature on the mammographic and sonographic appearance of breast cancer in *BRCA*-positive patients' reconstructed breasts is rather scanty. Pathologic studies have demonstrated that tumors in *BRCA1 *and *BRCA2 *mutation carriers are associated with morphologic features of continuous pushing margins [13], with a reduced potential for stromal infiltration explaining that this appearence might mimic benign-looking lesions at mammography [14] and breast sonography as well. Indeed, sonographic criteria of the mass in our case – ovoid axis, thin pseudocapsule, posterior enhancement, and well defined margins – were in keeping with a benign nodule [15]. In addition, according to Giovagnorio criteria [16], the lesion described in our case, with a single vascular pole (type 2), was compatible with a benign lesion. Cconsistent with the Lamb *et al *study [17], this lesion appeared benign but was in fact a moderate to high-grade invasive cancer.

## Conclusion

We report an unusual case of late occurrence of breast cancer after SCM in a *BRCA2 *mutation carrier. As these cases are so rare, the long-term risk of breast cancer following preventive mastectomy in *BRCA1/2*-positive individuals is likely to be very low. Nevertheless, vigilant, long-term surveillance based on clinical examination combined with breast sonography when indicated remains necessary, as delayed malignancy can occur.

## Competing interests

The author(s) declare that they have no competing interests.

## Authors' contributions

**LK **obtained family history, searched literature and drafted the manuscript, **BM **performed the sonographic assessment and assisted in manuscript preparation,**FT **initiated the report, provided patient history and referral for genetic counseling, managed the patient, MG carried out the molecular genetic studies, **FH **carried out the histopathological studies and provided diagnostic consultation, **WDF **obtained patient consent, assisted in literature search, helped to draft the manuscript and edited the final version. All authors read and approved the final manuscript.
